# Naturally Produced Lovastatin Modifies the Histology and Proteome Profile of Goat Skeletal Muscle

**DOI:** 10.3390/ani10010072

**Published:** 2019-12-31

**Authors:** Teik Kee Leo, Sani Garba, Danmaigoro Abubakar, Awis Qurni Sazili, Su Chui Len Candyrine, Mohammad Faseleh Jahromi, Yong Meng Goh, Ron Ronimus, Stefan Muetzel, Juan Boo Liang

**Affiliations:** 1 Institute of Tropical Agriculture and Food Security, Universiti Putra Malaysia, Serdang 43400, Malaysia; leoteikee@gmail.com (T.K.L.); sanigarba2003@yahoo.com (S.G.); awis@upm.edu.my (A.Q.S.); ymgoh@upm.edu.my (Y.M.G.); 2 Faculty of Veterinary Medicine, Universiti Putra Malaysia, Serdang 43400, Malaysia; abubakar.danmaigoro@udusok.edu.ng; 3 Faculty of Agriculture, Universiti Putra Malaysia, Serdang 43400, Malaysia; 4 Faculty of Sustainable Agriculture, Universiti Malaysia Sabah, Sandakan 90000, Malaysia; candyrine.suchuilen@ums.edu.my; 5 Agricultural Biotechnology Research Institute of Iran, Mashad 844, Iran; mfjahromi@yahoo.com; 6 Rumen Microbiology, AgResearch, Palmerston North 4442, New Zealand; Ron.Ronimus@agresearch.co.nz (R.R.); Stefan.Muetzel@agresearch.co.nz (S.M.)

**Keywords:** histology, lovastatin, methane, proteomics, skeletal muscle

## Abstract

**Simple Summary:**

Enteric methane formation in ruminants is one of the major contributors to climate change. Among the potential strategies, the supplementation of naturally produced lovastatin has been reported as one of the promising approaches for the mitigation of methane emissions. Nevertheless, statins have been associated with the development of muscle-related adverse effects which could affect the health and wellbeing of the animals. We have reported previously that supplementation of naturally produced lovastatin at 2 and 4 mg/kg body weight (BW), reduced methane emissions in goats without adversely affecting rumen fermentation and animal performance, except at higher level of lovastatin (6 mg/kg BW). However, the effects of lovastatin on the skeletal muscle in goats and the associated mechanisms have not been studied. Hence, the present study aimed to examine the effects of lovastatin on the histology of the goat skeletal muscle from the above study and to further elucidate the related underlying biochemistry processes. Histology analysis observed marked degeneration in the *longissimus thoracis et lumborum* muscle of goats supplemented with 6 mg lovastatin/kg BW. Our preliminary label-free proteomics analysis identified approximately 400 proteins in total, a number of which were differentially expressed, which are involved in energy metabolism and may have contributed to the observed skeletal muscle damage above 4 mg/kg BW.

**Abstract:**

This study was conducted to examine the effects of different levels of lovastatin on the histological and sarcoplasmic proteome profile of goat skeletal muscle. A total of 20 intact male Saanen goats were randomly assigned in equal numbers to four groups and fed a total mixed ration containing 50% rice straw, 22.8% concentrates and 27.2% of various proportions of untreated or treated palm kernel cake (PKC) to achieve the target daily intake levels of 0 (Control), 2 (Low), 4 (Medium) or 6 (High) mg lovastatin/kg BW. A histological examination discovered that the *longissimus thoracis et lumborum* muscle of animals from the Medium and High treatment groups showed abnormalities in terms of necrosis, degeneration, interstitial space and vacuolization. Our preliminary label-free proteomics analysis demonstrates that lovastatin supplementation induced complex modifications to the protein expression patterns of the skeletal muscle of the goat which were associated with the metabolism of carbohydrate and creatine, cell growth and development processes and other metabolic processes. The changes in these biochemical processes indicate perturbations in energy metabolism, which could play a major role in the development of myopathy. In conclusion, the present study suggests that supplementation of naturally produced lovastatin above 4 mg/kg BW could adversely affecting the health and wellbeing of treated animals.

## 1. Introduction

Methane is one of the major greenhouse gases contributing to climate change. Livestock production has been reported to contribute approximately 18% of global methane emissions and 9% of carbon dioxide production [1], which results primarily from the enteric fermentation of feeds [2]. Enteric methane formation results from the activity of complex interactions of anaerobic bacteria which, together, enable the degradation of ruminant feeds and methanogenic archaea which help remove metabolic hydrogen in the rumen [3]. Despite the importance of methanogenesis in maintaining low partial pressure of hydrogen required for efficient ruminal fermentation, the formation of methane also represents a 2–12% loss of gross dietary energy [4]. Hence, extensive research efforts are focused on the development of strategies to modify ruminal fermentation for reduction of methane emissions [5] as well as better feed utilization [6].

Among the potential strategies for mitigating methane emissions, supplementation of feed additives such as ionophores [7], organic acids [8], fatty acids [9], methyl coenzyme M reductase inhibitors [10], vaccine [11] and oils [12] have been extensively researched. However, many of these strategies have limited application due to their negative effect(s) on human and animal health, animal performance parameters and economical acceptance [13]. Supplementation of naturally produced lovastatin is a promising approach for mitigating methane emissions.

Lovastatin (C_24_H_36_O_5_, M.W. 404.55) is a secondary metabolite that is produced during the secondary phase (idiophase) of fungal growth. It is a competitive inhibitor of 3-hydroxy-3-methylglutaryl coenzyme A (HMG-CoA) reductase, which is a key enzyme in the cholesterol production pathway [14]. Inhibition of HMG-CoA reductase will mediate the suppression of cholesterol synthesis and cell membrane formation in methanogenic archaea. A previous study has shown that significant reduction in the growth and activity of methanogenic archaea using lovastatin without any negative effect on cellulolytic bacteria [15]. In addition, naturally produced lovastatin has been shown to mitigate methane gas emissions while simultaneously enhancing the digestibility of feed [16].

A previous study has reported the effects of naturally produced lovastatin from fermented-*Monascus purpureus* red rice powder on cattle [17]. A high dose of fermented-*Monascus purpureus* red rice powder (100 g/day and above) supplementation adversely affected dry matter intake and ruminant physiology. We have recently reported that supplementation of naturally produced lovastatin in goats as being capable of mitigating methane emissions effectively without adversely affecting digestion and rumen fermentation, except that animals fed the highest level (6 mg/kg BW) had lower appetite [18].

Statins are a class of clinically important 3-hydroxy-3-methylglutaryl-coenyme A (HMG-CoA) reductase inhibitors that are widely used in humans for the prevention and treatment of cardiovascular disease [19]. One main recognized side effect of the use of stains is ‘statin-induced myotoxicity’ [19,20]. Various hypotheses have been suggested to be the possible mechanisms behind statin-induced myopathy, including the alteration of the muscle cell membrane function due to the impairment of cholesterol synthesis, adverse effects on energy metabolism, including the production of ATP, and effects on the master energy regulator AMPK (adenosine-monophosphate kinase) [19], the depletion of intermediates of the cholesterol synthesis pathway that has secondary effects on small regulatory GTP-binding proteins and the reduction of the levels of ubiquinone (the latter which would negatively affect mitochondrial energy metabolism), altered calcium metabolism, direct effects on sarcoplasma lactate and chloride levels, and effects on muscle cell apoptosis (and muscle remodeling) [20]. However, the precise mechanisms have not been elucidated and the effects of lovastatin on the skeletal muscle in goats have not been studied. Therefore, this follow-up study was conducted to examine the effects of lovastatin on the histology of the goat skeletal muscle from the above study to further elucidate whether supplementation of lovastatin affects the health and wellbeing of the goats. In addition, a label-free proteomics approach was utilized to illuminate the underlying biochemical processes in the goat skeletal muscle.

## 2. Materials and Methods

### 2.1. Animals and Management

This study was approved by the Animal Care and Ethics Committee of the Universiti Putra Malaysia (UPM/IACUC/AUP-R0087/2015). Detailed protocols of the study have been reported [18]. Briefly, twenty intact male Saanen goats of 4–5 months old with an average live weight of 26 ± 3.4 kg were used in the 12-week feeding trial. The animals were randomly assigned in equal numbers and fed a total mixed ration containing 50% rice straw, 22.8% concentrates and 27.2% of various proportions of untreated or treated PKC to achieve the target daily intake level of 0 (Control), 2 (Low), 4 (Medium) or 6 (High) mg lovastatin/kg BW. The lovastatin was produced by solid state fermentation using PKC (palm kernel cake) and *Aspergillus terreus* (ATCC 74135) [18].

### 2.2. Slaughtering and Sample Collection

At the end of the feeding trial, the goats were slaughtered according to a halal slaughtering procedure as outlined in the MS1500: 2009 [21]. The process involved severing the carotid arteries, jugular veins, trachea and esophagus. The neck cut position was performed at the level of the first cervical vertebra (C1) based on the requirements of OIE [22]. Immediately after skin removal and evisceration (within 15 min postmortem), without any application of carcass electrical stimulation, *longissimus thoracis et lumborum* muscle was collected from each animal. The muscle samples collected for histology analysis were rinsed with a normal saline solution before fixing in 10% PBS-buffered formalin solution. For the proteome profile, pre-rigor muscle samples collected at 15 min postmortem, were snap frozen in liquid nitrogen and kept at −80 °C until subsequent analysis.

### 2.3. Histology

*Longissimus thoracis et lumborum* muscle samples were removed from the formalin solution, dehydrated in an increasing ethanol series and routinely processed for paraffin embedding. The samples were sectioned at 5 µm and stained with haematoxilin-eosin. From each muscle, two locations were sectioned and each location was mounted on a slide and viewed with a Leica DM LB2 upright light microscope (Leica microsystems Wetzlar GmbH, Wetzlar, Germany). Images were captured from each slide under 20× magnification using a Leica DFC320 digital camera connected to a computer which was controlled with Leica IM50 v4.0 software (Leica microsystems Wetzlar GmbH, Wetzlar, Germany). In total, ten slides for each treatment group were examined.

The muscle tissues were evaluated for evidence of necrosis, degeneration, interstitial space and vacuolization. Numerical scores were assigned based on degree of severity (0 = normal to 5 = marked) according to Gall et al. [23] (Table 1). The measurement of muscle fiber diameter was conducted according to the procedure described by Sandri et al. [24]. Briefly, muscle fiber size at × 100 from four random fields were utilized on the photomicrograph with Motic camera software (Motic Images Plus 3.0, Hong Kong, China) and values were evaluated using four replications per treatment (n = 16).

A statistical analysis was conducted using Statistical Analysis System (SAS) package version 9.2 software (Statistical Analysis System, 2007, SAS Institute Inc., Cary, NC, USA). Histological scores of necrosis, degeneration, interstitial space and vacuolization between every treatment group were compared using the Kruskal–Wallis test. The data of the muscle fiber diameter were analyzed using the General Linear Model (GLM) procedure, and significant differences between means were separated using Duncan’s multiple range tests. Statistical confidence was considered as *p* < 0.05.

### 2.4. Liquid Chromatography Mass Spectrometry

Crude protein was extracted from each muscle sample. Briefly, 0.2 g of muscle sample in powder form was mixed with 1 mL of cold buffer containing 100 mm Tris, pH 8.3 and 10 µL protease inhibitor (Calbiochem^®^). The samples were mixed thoroughly with vortex for 30 s and centrifuged at 4 °C for 20 min at 15,000 *g*. The supernatants were carefully collected and the concentrations were determined using the Bradford assay [25].

Each protein sample (100 mg) was reduced with 50 mm DTT at 60 °C for 60 min and alkylated with 50 mm iodoacetamide in the dark for 45 min at room temperature. Then, proteins were diluted with 50 mm ammonium bicarbonate and digested with trypsin at 37 °C overnight. The digestion process was stopped by adding 0.5% formic acid. Digested peptides were desalted using C18 ZipTip pipette tips (Millipore, Billerica, MA, USA) according to the supplier’s instructions and resuspended in 0.1% formic acid.

The purified digested peptides were separated with reverse phase liquid chromatography using a Dionex Ultimate 3000 RSLCnano system (Thermo Fisher Scientific Inc., Waltham, MA, USA) and analyzed by tandem mass spectrometry using an Orbitrap Fusion mass spectrometry (Thermo Fisher Scientific Inc., Waltham, MA, USA.). Peptide samples (2 µL) were separated on the EASY-Spray Column Acclaim PepMapTM C18 100 Å (2 µm particle size, 50 µm id × 25 cm; Thermo Fisher Scientific Inc., Waltham, MA, USA.) by a gradient from 5% to 40% of buffer B (0.1% formic acid in acetonitrile) at 300 nL/min flow over 91 min. The remaining peptides were eluted by a short gradient (2 min) from 40% to 95% buffer B.

The eluting peptides were analyzed using tandem mass spectrometry using Orbitrap Fusion mass spectrometry. Full scan spectra were collected using the following parameters: scan range 310–1800 m/z, resolving power of 120,000, AGC target of 400,000, and maximum injection time of 50 ms. The method consisted of a 3 s Top Speed Mode where precursors were selected for a maximum 3 s cycle. Only precursors with an assigned monoisotopic m/z and a charge state of 2–7 were further analyzed for MS2. All the precursors were filtered using a 20 s dynamic exclusion window and intensity threshold of 5000. The MS2 spectra were analyzed using the following parameters: rapid scan rate with a resolving power of 60,000, AGC target of 100, 1.6 m/z isolation window, and a maximum injection time of 250 ms. Precursors were fragmented by CID and HCD at normalized collision energy of 30% and 28%.

The raw data obtained were analyzed using Thermo ScientificTM Proteome Discoverer^TM^ Software Version 2.1 (Thermo Fisher Scientific Inc., Waltham, MA, USA) by searching a goat (*Capra hircus*) database and mammalian database downloaded from UniProt. The parameters for searching were set as follows: missed cleavage: 2; MS1 tolerance: 10 ppm; MS2 tolerance: 0.6 Da; variable modification: oxidation (M), deamidation of asparagine (N) and glutamine (Q); and fixed modification: carbamidomethyl (C). All the peptides were validated using the Percolator© algorithm based on q-value less than 1% false discovery rate.

A quantitative analysis of the data was performed using Perseus version 1.6.8.0 to identify the differentially expressed proteins in the muscle between the treatment groups. Pair-wise comparisons between each group were conducted using two-tailed Student *t*-tests. Statistical significance was set at *p* < 0.05. A gene ontology enrichment analysis and functional annotation of the identified proteins were performed using the Gene Ontology database through the Panther Classification System and Database for Annotation, Visualization and Integrated Discovery (DAVID) version 6.8 (https://david.ncifcrf.gov) [26]. The interaction of the differentially expressed proteins was constructed by String v11.0.

## 3. Results

### 3.1. Histology

**Figure 1:**
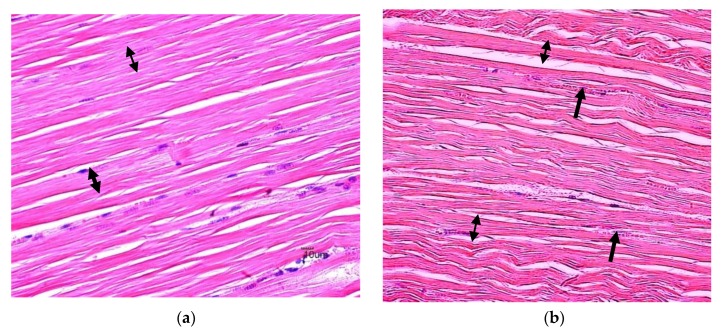

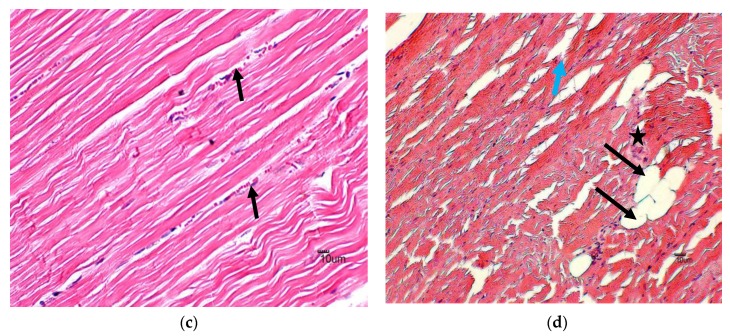
Histological analysis on *longissimus thoracis et lumborum* muscle of goats supplemented with naturally produced lovastatin (**a**) A photomicrograph of longitudinal section of muscle from control (0 mg/kg BW) group showing normal morphological architecture of the skeletal muscles, with myofibril and myocyte nuclei located peripherally on the myofibril filaments with myofibril separated by interstitial space and the striated character of normal muscular structure (double-ended arrow) (H&E × 400). (**b**) A photomicrograph of longitudinal section of muscle (2 mg lovastatin/kg LW) showing mild hemorrhages within the interstitial space (arrow) and striated character of normal muscle structure (double-ended arrow) (H&E × 400). (**c**) Photomicrograph of muscle (4 mg lovastatin/kg LW) showing mild hemorrhages within the interstitial space with normal morphology myofilament (H&E × 400) (**d**) Photomicrograph of muscle (6 mg lovastatin/kg LW) showing marked myofibrillar degeneration (star), cellular infiltration associated with myofibril rupture (blue arrow) and vacuolization of myocytes and interstitial space (arrow) (H&E × 400).

The histological examinations showed that the skeletal muscle of animals supplemented with lovastatin displayed light to moderate tissue degeneration (Figure 1). Mild haemorrhage was observed in the Medium treatment group while skeletal muscle of the High treatment group showed relatively severe degeneration as compared to the other treatment groups. Descriptive data for each group is shown in Table 2. The Kruskal–Wallis test showed a significant difference among the four groups. It was observed that the muscle of the Control group was normal, with no signs of any degeneration. The high treatment group had the highest (*p* < 0.05) score of degeneration, while the scores of Low and Medium treatment groups were significantly higher (*p* < 0.05) than the Control group. The score of necrosis was similar between Control and Low treatment groups, but significantly higher (*p* < 0.05) in the Medium treatment group, and highest (*p* < 0.05) in the High treatment group. For both interstitial space and vacuolization, the scores of the Medium and High treatment groups were similar and significantly higher (*p* < 0.05) than the Control and Low treatment groups. There were insignificant differences in the scores of interstitial spaces and vacuolization between the Control and Low treatment groups.

**Figure 2:**
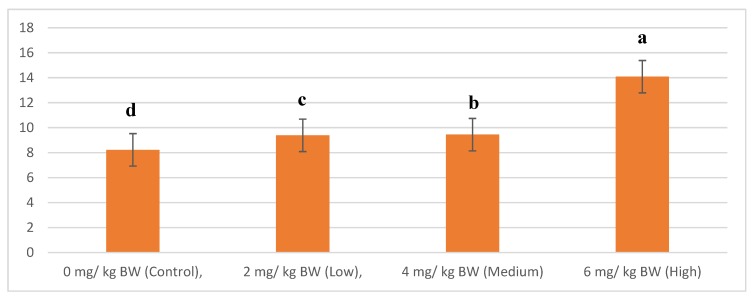
The fiber diameter (mm) of *longissimus thoracis et lumborum* muscle of goats supplemented with naturally produced lovastatin. ^a,b,c,d^ Score within a row with different superscripts differ significantly at *p* < 0.05.

The fiber diameter results indicate that significant differences were present between the treatment groups (*p* < 0.05). As shown in Figure 2, muscle fiber diameter increases with increase in lovastatin concentration. The Control group recorded the lowest fiber diameter (8.22 mm) while the High treatment group recorded the highest fiber diameter (14.08 mm).

### 3.2. Differentially Expressed Proteins

**Figure 3:**
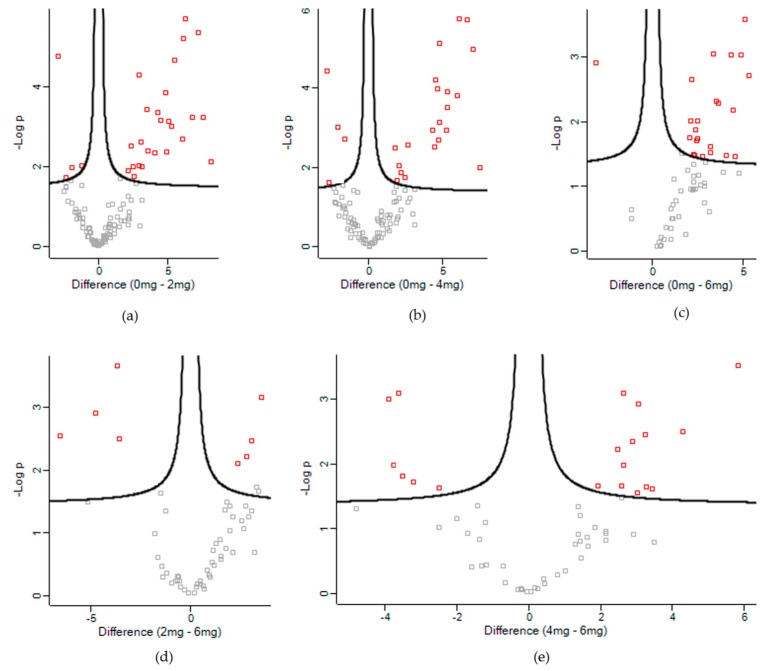
Volcano plots of differentially expressed proteins in: (**a**) Control vs. Low, (**b**) Control vs. Medium, (**c**) Control vs. High, (**d**) Low vs. High and (**e**) Medium vs. High.

**Figure 4:**
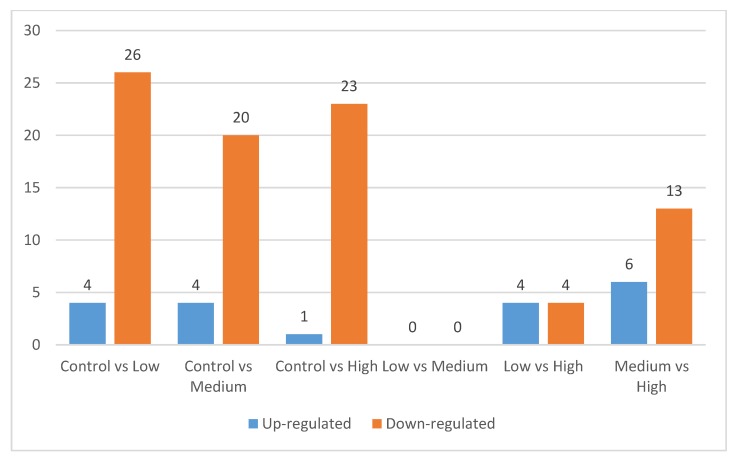
Number of differentially expressed proteins in six comparisons: Control vs. Low, Control vs. Medium, Control vs. High, Low vs. Medium, Low vs. High and Medium vs. High.

The present study identified approximately 400 proteins in the *longissimus thoracis et lumborum* muscle of goat. In order to access the dynamic changes of proteins following supplementation of lovastatin, comparisons were made between the control and lovastatin-treated groups, as well as between the lovastatin-treated groups (Figure 3). Figure 4 shows the number of proteins that were differentially expressed when compared among the treatment groups. Comparisons between control and lovastatin-treated groups demonstrated that lovastatin supplementation induced complex modifications to the protein expression patterns in the *longissimus thoracis et lumborum* muscle of the goat (Supplementary Materials Figure S1). When the Low treatment and Control groups were compared, there were 26 proteins down-regulated and four proteins up-regulated in the Low treatment group. When the Medium treatment and Control groups were compared, 20 proteins were observed to be down-regulated and four proteins up-regulated. When the High and Control treatment groups were compared, 23 proteins were down-regulated and one protein was up-regulated in the muscle tissue. Differentially expressed proteins were also identified when the proteome profile of the High treatment group was compared with Low and Medium treatment groups. When compared with the Low treatment group, four proteins were up-regulated and four were down-regulated in the High treatment group. When the High and Medium treatment groups were compared, 13 proteins were down-regulated and six proteins were up-regulated. However, no proteins were differentially expressed when the Low and Medium treatment groups were compared. Based on the *p*-value, the top 10 differentially expressed proteins of each comparison group are shown in Table 3.

**Figure 5:**
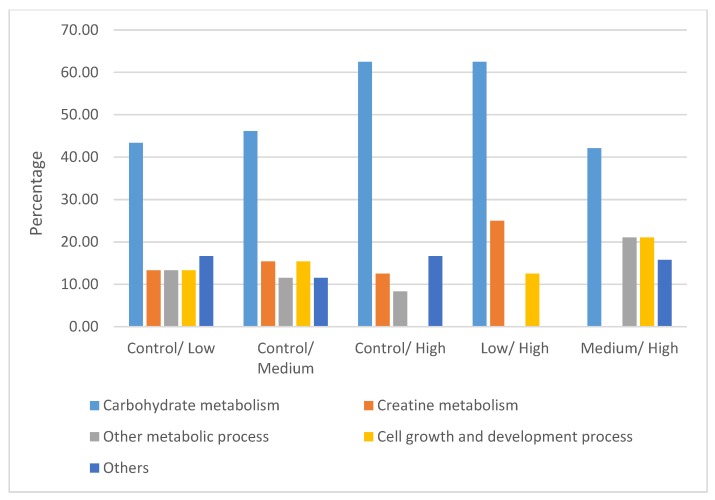
Classifications of differentially expressed proteins according to the functional roles in the following categories: carbohydrate metabolism, creatine metabolism, other metabolic process, cell growth and development process and others.

**Figure 6:**
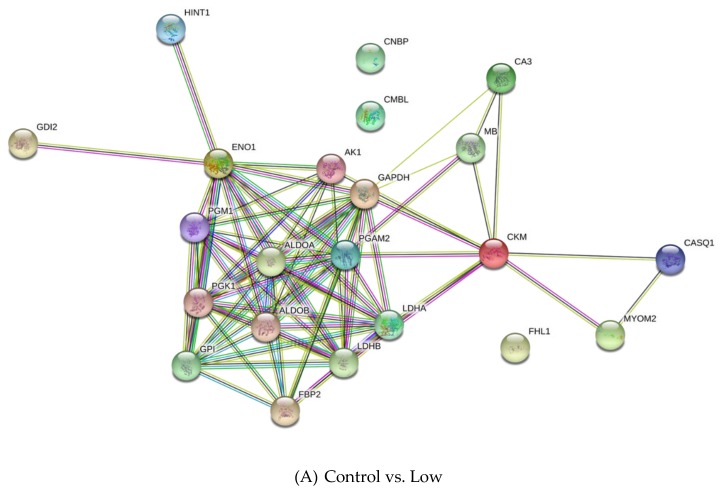

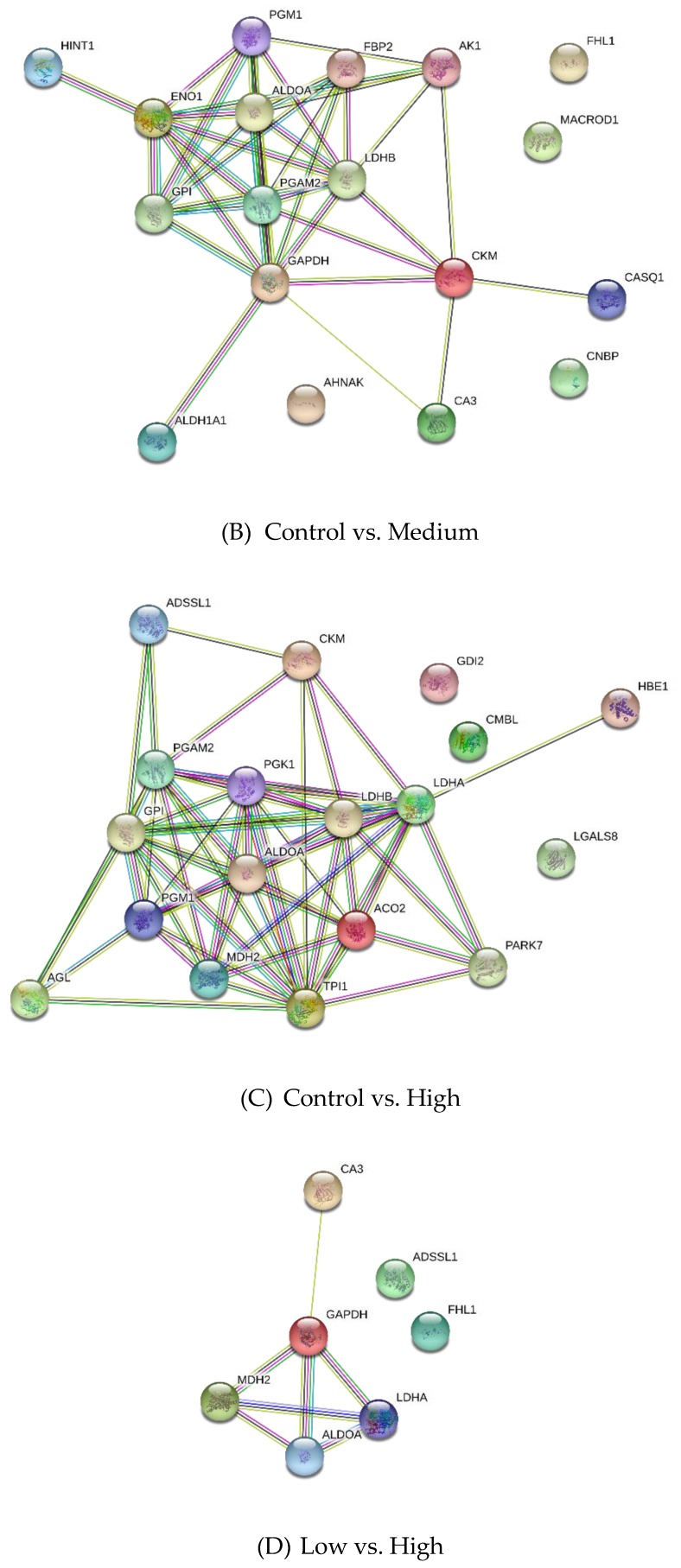

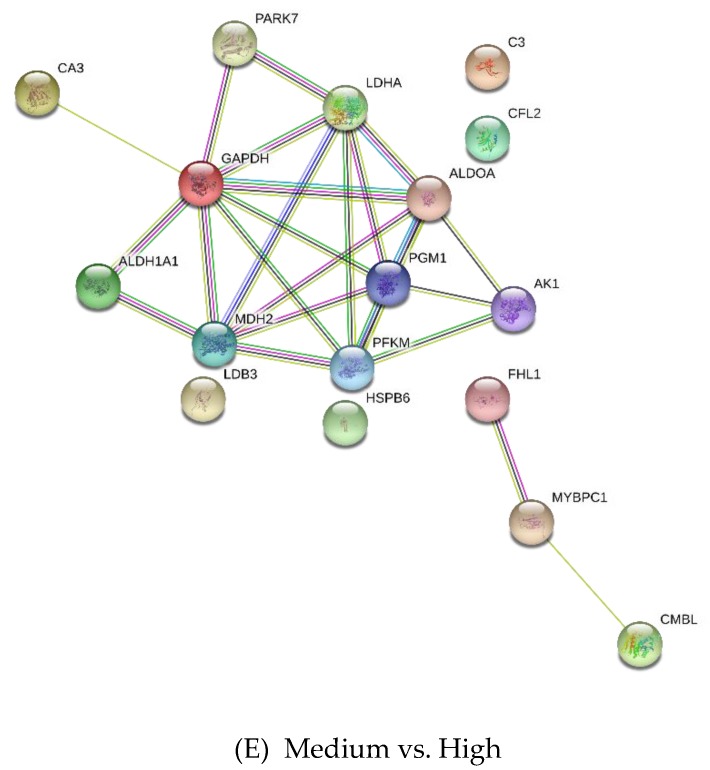
The protein–protein interaction network of differentially expressed proteins in the comparisons: Control vs. Low (**A**), Control vs. Medium (**B**), Control vs. High (**C**), Low vs. High (**D**) and Medium vs. High (**E**). Colored ball: the changed protein; yellow line: text mining; purple line: experiments; blue line: databases; light blue: homology; black line: co-expression; green line: neighborhood; red line, gene fusion; deep blue line: cooccurrence.

The differentially expressed proteins were classified using the Panther Classification System and DAVID. These proteins were grouped on the basis of their functional role in the following categories: carbohydrate metabolism, creatine metabolism, other metabolic processes, cell growth and development process, and others (Figure 5). The interaction networks of the differentially expressed proteins were generated using String v11.0 and are presented in Figure 6. Generally, these proteins are involved in a network of carbohydrate metabolism, energy production, and skeletal and muscular system development and function.

## 4. Discussion

Statins are the most widely used lipid lowering agents and act by inhibiting HMG-CoA reductase in the cholesterol biosynthesis pathway. Nevertheless, the use of statins is reported to have adverse side effects, such as muscular pain, cramps and/or stiffness on skeletal muscles in humans [27]. We had previously reported that lovastatin effectively decreased methane production in goats [18]. Given the potential for myopathies at high therapeutic doses, the effects of naturally produced statins on the skeletal muscles of ruminants was a primary interest of ours. Hence, the present study examined the effects of naturally produced lovastatin on the histology and proteome profile of the representative goat skeletal muscle *longissimus thoracis et lumborum*.

### 4.1. Histology

*Longissimus thoracis et lumborum* muscle tissues were stained with haematoxilin-eosin and evaluated for evidence of necrosis, degeneration, interstitial space and vacuolization. The present study shows that supplementation of lovastatin was associated with marked adverse effects on the muscle. The dose levels of the lovastatin supplementation are positively correlated to the extent of cellular damage on the skeletal muscle as reported previously [28]. The supplementation of 2 mg lovastatin/kg BW induced a low but noticeable degeneration of the muscle fiber in the goats. At higher dosages (4 and 6 mg/kg), naturally produced statin resulted in a higher degree of necrosis and degeneration, as well as larger interstitial spaces and vacuolization in the skeletal muscle.

The present study shows that the muscle fiber diameter in goats increases with the increase in lovastatin concentration. This could be attributed to the biological effort to repair or replace damaged muscle fibers. Statins are known to impair cholesterol synthesis by inhibiting the production of mevalonate and ubiquinone in the cholesterol biosynthetic pathway, thus preventing oxidative phosphorylation within mitochondrial membrane by prenylation, resulting in an increase in muscle fiber diameter and the reduction of atrogin-1 expression [29]. However, the aberrant organelles observed throughout the sarcoplasm with concentric myelinoid bodies on muscles ultrastructure treated with rosuvastatin in the study of Westwood et al. [30], without any significant notable ultrastructure change could be linked to the increase muscle fiber diameter recorded. This initiates with the satellite cells fusing together, thus leading to increases in muscle fiber cross-sectional area or hypertrophy. Muscle fiber size increases have been reported previously in sheep fed Cimaterol [31] as well as beef cattle fed ractopamine hydrochloride and zilpaterol hydrochloride [32]. In addition, Hanai et al. [33] reported that lovastatin leads to clear dose-dependence of statin-induced muscle damage using Zebra fish as the animal model. The present results are in agreement with earlier studies that statin-induced myopathy is dose-related [34,35,36].

### 4.2. Differentially Expressed Proteins

Label-free proteomics analysis via LCMS demonstrated that the supplementation of lovastatin induced modifications on the expression of a number of proteins, regardless the concentration of lovastatin. These data suggest that lovastatin had an effect on a wide range of biological functions in the muscle. Lovastatin supplementation impaired the energy production system in the skeletal muscle, particularly in the metabolism of carbohydrate and creatine. Similar observations have been reported on the *extensor digitorum longus* muscle of rats treated for 2 months with 10 mg atorvastatin/kg BW and 20 mg fluvastatin/kg BW [20]. The impairment in the energy production system could play a major role in the development of muscle damage, which is consistent with the adverse effects observed on the *longissimus thoracis et lumborum* muscle through histological examination.

#### 4.2.1. Carbohydrate Metabolism

The present study shows that lovastatin supplementation down-regulated proteins involved in glycolysis, gluconeogenesis and the tricarboxylic acid (TCA) cycle. Glycolysis is an oxygen-independent pathway that converts 6-carbon glucose into pyruvate. Through this metabolic process, high-energy adenosine triphosphate (ATP) molecules and reduced nicotinamide adenine dinucleotide (NADH) are generated. Glycolytic enzymes such as alpha-enolase, fructose-bisphosphate aldolases, glucose-6-phosphate isomerase, glyceraldehyde-3-phosphate dehydrogenase, phosphoglycerate kinase 1 and phosphoglycerate mutase 2 were down-regulated following lovastatin supplementation. In addition, phosphoglucomutase-1, that catalyzes the bi-directional inter-conversion of glucose-1-phosphate and glucose-6-phosphate, was also down-regulated following lovastatin supplementation. Glucose-1-phosphate is a substrate for the synthesis of UDP-glucose used to synthesis a variety of cellular constituents, while glucose-6-phosphate is the first intermediate in glycolysis. Similar observations of down-regulation of glycolytic enzymes in the skeletal muscle have also been reported previously in rat and the down-regulation of glycolytic enzymes is a symptom of energy production failure and can contribute to muscle damage [20]. In humans, hereditary muscle glycogenoses are characterized by defective glycolytic enzymes and lead to different degrees of myopathy [37].

The TCA cycle is the one of the most efficient ways of generating energy in aerobic organisms. Mitochondrial malate dehydrogenase, which was down-regulated in the High treatment group when compared to the Control, Low and Medium treatment groups, is an integral component of the TCA cycle. Malate dehydrogenase reversibly catalyzes the oxidation of malate to oxaloacetate that can be utilized in the TCA cycle and amino acid production [38]. Down-regulation of this enzyme indicated that a high level of lovastatin supplementation (6 mg/kg BW) results in the impairment of mitochondrial function, which is in agreement with the study by Päivä et al. [39] which reported a reduction of mitochondria volume in skeletal muscle following aggressive statin treatment.

#### 4.2.2. Creatine Metabolism

Generally, statin supplementation is associated with a higher concentration of creatine kinase in the blood plasma. The present study observed a down-regulation of creatine kinase following lovastatin supplementation. Similarly, a decrease of creatine kinase was also observed in the skeletal muscle in rats [20]. Creatine kinase is an enzyme that catalyzes the conversion of creatine to phosphocreatine by utilizing ATP. This enzyme also catalyzes the reverse reaction to produce phosphocreatine and ATP. In tissues utilizing a large amount of ATP such as skeletal muscle, the creatine kinase/phosphocreatine system plays a complex and multi-faceted role in the regulation of cellular energy homeostasis [40]. The ATP regeneration capacity of creatine kinase is very high and considerably exceeds both cellular utilization and replenishment through glycolysis and oxidative phosphorylation [40]. Interestingly, a previous study showed that transgenic mice lacking either the cytoplasmic or mitochondrial creatine kinase may develop muscle atrophy [41]. Together with proteins involved in carbohydrate metabolism, the down-regulation of creatine kinase indicated an impairment to the energy production system, which may lead to statin myopathy.

#### 4.2.3. Other Metabolic Processes

The down-regulation of adenylate kinase isozyme 1, adenylosuccinate synthethase isozyme 1 and carbonic anhydrase 3 may impair the energy production in the skeletal muscle. Adenylate kinase isozyme 1 and adenylosuccinate synthethase isozyme 1 play an important role in cellular energy homeostasis and more specifically, in adenine nucleotide metabolism. Adenylate kinase isozyme 1 catalyzes the reversible transfer of phosphate between ATP and adenosine monophosphate (AMP), while adenylosuccinate synthethase isozyme 1 interconverts inosine monophosphate (IMP) and AMP to regulate nucleotide levels in the tissue, and which contributes to the regulation of glycolysis. Meanwhile, the lack of carbonic anhydrase 3 is suggested to impair mitochondrial ATP synthesis in the gastrocnemius muscle of rat [42]. Furthermore, carbonic anhydrase 3 is also shown to provide protection to the cells against free radicals [43]. The higher level of carbonic anhydrase 3 in the High treatment group when compared to Low and Medium treatment groups, may be due to its role in cell protection against oxidative stress.

Retinal dehydrogenase 1, glycerol-3-phosphate dehydrogenase [NAD^+^], L-lactate dehydrogenase A chain and L-lactate dehydrogenase B chain are involved in redox cofactor metabolism, which plays a central role in meeting cellular redox requirements of proliferating mammalian cells. Retinal dehydrogenase 1 converts retinaldehyde to retinoic acid, which directly catalyzes the regeneration of NADH. Glycerol-3-phosphate dehydrogenase catalyzes the reversible conversion of dihydroxyacetone phosphate to glycerol-3-phosphate, which involves the redox reaction of NADH and NAD^+^. Meanwhile, lactate dehydrogenase catalyzes the conversion of pyruvate into lactate. Usually, a large amount of lactate is generated in proliferating cells to allow high glycolytic flux to support the generation of ATP and biosynthetic precursors [44]. At the same time, the generation of lactate also involves the conversion of NADH to NAD^+^ by lactate dehydrogenase. NAD^+^ is crucial as it is directly used to oxidize precursors of some nucleotides and amino acids and many intermediates of NAD^+^-dependent pathway are important precursors for biosynthesis [44]. A reduction in these proteins may affect the NADPH-dependent pathways, thus blocking the ATP production pathways.

#### 4.2.4. Cell Growth and Development Process

The present findings also identified a number of differentially expressed proteins (including myomesin-2, cellular nucleic acid binding protein, FHL1 (four and a half LIM domains protein 1), myosin-binding protein C, cofilin-2 and calsequestrin-1) associated with structural muscle proteins and muscle health. Myomesin-2 is a major component of myofibrillar M band that forms a network at the center of the sarcomere to anchor thick filaments within the A band. Prill et al. [45] observed an increased myomesin1a expression at the stage of thick filament assembly in zebrafish myosin chaperone mutants, which signifies a myomesin-dependent response pathway to sarcomere damage at the earliest stages of muscle disease. Up-regulation of myomesin-2 in the Low treatment group may be an early indication of sarcomere damage responding to the supplementation of lovastatin.

The deficiency of myosin-binding protein C and cofilin-2 in the present study may indicate the development of myopathy following the high lovastatin supplementation (6 mg/kg). Myosin-binding protein C is a myosin-associated protein found in the cross-bridge bearing zone of A bands in striated muscle that regulates thin filament activity in a Ca^2+^-dependent manner [46]. This protein plays an important role in muscle contraction by recruiting muscle-type creatine kinase to myosin filaments. Cofilin-2 is one of the three proteins of the actin-depolymerization factor (ADF)/cofilin family that bind to F-actin, increase F-actin torsional dynamics and subsequently sever F-actin [47]. Deficiency of cofilin-2 is associated with myopathy and may result in a reduced polymerization of actin filament, causing their accumulation in nemaline bodies, minicores and concentric laminated bodies [48].

The reduction in the expression of calsequestrin and FHL1, that are involved in muscle development, was associated with the muscle damage. Calsequestrin is a Ca^2+^-binding protein, which has been showed to be decreased in dystrophic mouse skeletal muscle [49], while mutation in the FHL1 gene is associated with myopathy [49]. FHL1 is a multifunctional protein likely to be involved in ion channel binding and muscle development.

Recently, it has been reported that the modifications of the cellular nucleic acid binding protein, which are indicated to play a role in myotonic dystrophy type 2 disease, may result in muscle atrophy through affecting myofiber membrane function [50]. This protein was down-regulated in the Low treatment group but up-regulated in the Medium treatment group when compared to the Control group. The mechanism of cellular nucleic acid binding protein associated with lovastatin induced myopathy is yet to be established. Together with Rab GDP dissociation inhibitor beta which involved in the regulation of vesicle-mediated cellular transport [51], the regulation of both proteins in the present study indicated that the mechanism associated with tissue regeneration or repair was activated in the muscle tissue.

#### 4.2.5. Other Proteins

In addition to the energy production system, proteins involved in other cellular processes were also affected by lovastatin. Proteins such as galectins and Protein DJ-1, which are in involved in the regulation of the apoptotic pathway, were also down-regulated following lovastatin supplementation. Galectins have a diverse range of biological functions including regulation of pre-mRNA splicing, cell adhesion, cell growth, differentiation, apoptosis and cell cycle [52], while protein DJ-1 plays an important role in cell protection against oxidative stress and cell death [53].

Furthermore, transport proteins such as beta A globin chain and myoglobin were also down-regulated following lovastatin supplementation. Globins are small globular metalloproteins containing a heme prosthetic group by which they can reversibly bind to gaseous ligands such as oxygen and carbon dioxide. In skeletal muscle, myoglobin is the most important globin functioning as a local store and mobile carrier of oxygen in response to the mitochondrial demand [54]. The down-regulation of globins may further impact the mitochondrial functions and skeletal muscle damage.

Overall, the present study shows that lovastatin supplementation down-regulates proteins involved in the energy production system (particularly the glycolytic pathway and creatine metabolism), regardless of the concentration of lovastatin. Supplementation with a high concentration of lovastatin (6 mg/kg BW) could further impair the mitochondria and thus the TCA cycle. Moreover, supplementation of lovastatin could also affect skeletal muscle development through the ion channel. At the same time, the present study also observed the activation of tissue regeneration or repair in the muscle tissue following the supplementation of lovastatin. Furthermore, changes in the expression of proteins involved in apoptosis and oxidative damage suggests an accentuated sensitivity of statin-treated muscle to oxidative stress. Oxidative stress can promote increased proteolysis and depress protein synthesis and trigger many conditions associated with muscle wasting [55]. Such perturbation in energy metabolism and ATP synthesis may have profound effects on protein synthesis and contribute to metabolic stress, which could play a major role in the development of myopathy.

The present study is the first study to report the preliminary findings on the effects of different levels of naturally produced lovastatin on the histology and proteome profile of *longissimus thoracis et lumborum* muscle of goats. As skeletal muscle is versatile and comprises of a large variety of functionally diverse fiber types, future analyses taking the muscle fiber phenotype into account would provide further information in relation to myopathy as affected by lovastatin. In addition, the proteome of skeletal muscle is highly complex and dynamic, hence, further validation is required to strengthen the observed modifications on the biochemical metabolisms affected by lovastatin. Furthermore, a study on the global metabolite profile in the blood and skeletal muscle tissue would complement the global protein expression profile established in the present study. The integration between proteomics and metabolomics profiles would provide a more holistic overview of the biochemical changes that occurred.

## 5. Conclusions

The histology scores indicate increasing muscle damage to the *longissimus thoracis et lumborum* muscle of goats supplemented with increasing dosages, particularly at 6 mg/kg BW, due to naturally produced lovastatin. The proteomics analysis revealed that lovastatin supplementation induced complex modifications to carbohydrate metabolism, energy production, and skeletal and muscular system development of skeletal muscle of goats, which may have contributed to the observed skeletal muscle damage. Taken together, it is clear that supplementation of naturally produced lovastatin above 4 mg/kg BW is too high, which can adversely affect the health and wellbeing of the animals.

## Figures and Tables

**Table 1:** Scoring system used in histological analysis on *longissimus thoracis et lumborum* muscle of goats supplemented with naturally produced lovastatin.

Score	Histopathologic Injury	Percentage
0	Normal	0%
1	Minimal	<15%
2	Mild	≤25%
3	Moderate	≤40%
4	Marked	≥50

**Table 2:** Histology scores of *longissimus thoracis et lumborum* muscle of goats supplemented with naturally produced lovastatin.

Parameters	Control			Low			Medium			High		
	Median	Min	Max	Median	Min	Max	Median	Min	Max	Median	Min	Max
Necrosis	0 ^a^	0	0	0 ^a^	0	0	1 ^b^	0	2	3	1	4
Degeneration	0 ^a^	0	0	0 ^b^	0	1	0 ^b^	0	2	2	0	3
Interstitial space	0 ^a^	0	0	0 ^a^	0	0	1 ^b^	0	3	1 ^b^	0	3
Vacuolization	0 ^a^	0	0	0 ^a^	0	0	1 ^b^	0	2	1 ^b^	0	3

Control, Low, Medium and High represent 0, 2, 4 and 6 mg lovastatin/kg BW, respectively. Data was presented as the median, minimum and maximum of the score. ^a,b^ Score within a row with different superscripts differ significantly at *p* < 0.05.

**Table 3:** Top 10 differentially expressed proteins in the Control vs. Low, Control vs. Medium, Control vs. High, Low vs. High and Medium vs. High groups.

UniProt Accession	Description	-Log *p*-Value	Difference ^1^
Control vs. Low			
Q9XSC6	Creatine kinase M-type	5.68	−6.27
P06733	Alpha-enolase	5.20	−6.08
P54296	Myomesin-2	4.75	2.93
P00559	Phosphoglycerate kinase 1	4.66	−5.45
P62633	Cellular nucleic acid-binding protein	4.28	−2.88
P02191	Myoglobin	3.83	−4.87
Q5S1S4	Carbonic anhydrase 3	3.42	−3.45
P16290	Phosphoglycerate mutase 2	3.34	−4.27
Q5E9B1	L-lactate dehydrogenase B chain	3.22	−7.52
P04406	Glyceraldehyde-3-phosphate dehydrogenase	3.22	−6.75
Control vs. Medium			
P15259	Phosphoglycerate mutase 2	5.72	−6.11
P06733	Alpha-enolase	5.72	−6.66
P05065	Fructose-bisphosphate aldolase A	5.10	−4.79
P36871	Phosphoglucomutase-1	4.95	−7.02
P62633	Cellular nucleic acid-binding protein	4.43	2.80
P06732	Creatine kinase M-type	4.20	−4.55
Q13642	Four and a half LIM domains protein 1	3.97	−4.63
P06744	Glucose-6-phosphate isomerase	3.90	−5.34
P00571	Adenylate kinase isoenzyme 1	3.49	−5.31
P04406	Glyceraldehyde-3-phosphate dehydrogenase	3.12	−4.77
Control vs. High			
P19858	L-lactate dehydrogenase A chain	3.57	−5.10
P13707	Glycerol-3-phosphate dehydrogenase [NAD(^+^)], cytoplasmic	3.05	−3.36
Q1KZF3	Beta A globin chain	3.04	−4.30
Q96DG6	Carboxymethylenebutenolidase homolog	3.04	−4.85
P50397	Rab GDP dissociation inhibitor beta	2.91	3.13
P36871	Phosphoglucomutase-1	2.71	−5.31
I1 × 3V1	Galectin	2.65	−2.16
P00559	Phosphoglycerate kinase 1	2.32	−3.53
Q5E9B1	L-lactate dehydrogenase B chain	2.18	−4.43
P06732	Creatine kinase M-type	2.02	−2.50
Low vs. High			
Q5S1S4	Carbonic anhydrase 3	3.65	3.63
P19858	L-lactate dehydrogenase A chain	3.15	−3.54
P00883	Fructose-bisphosphate aldolase A	2.91	4.71
P04406	Glyceraldehyde-3-phosphate dehydrogenase	2.54	6.5
Q13642	Four and a half LIM domains protein 1	2.5	3.55
K0J107	Malate dehydrogenase, mitochondrial	2.47	−3.06
A4Z6H0	Adenylosuccinate synthetase isozyme 1	2.21	−2.8
P13707	Glycerol-3-phosphate dehydrogenase [NAD(^+^)], cytoplasmic	2.11	−2.36
Medium vs. High			
Q96DG6	Carboxymethylenebutenolidase homolog	3.53	−5.82
Q5S1S4	Carbonic anhydrase 3	3.09	3.63
Q00872	Myosin-binding protein C, slow-type	3.09	−2.64
Q13642	Four and a half LIM domains protein 1	3.01	3.9
K0J107	Malate dehydrogenase, mitochondrial	2.93	−3.06
P48644	Retinal dehydrogenase 1	2.5	−4.3
P19858	L-lactate dehydrogenase A chain	2.46	−3.26
Q148F1	Cofilin-2	2.35	−2.89
P13707	Glycerol-3-phosphate dehydrogenase [NAD(^+^)], cytoplasmic	2.23	−2.49
Q99497	Protein DJ-1	1.99	−2.63

Control, Low, Medium and High represent 0, 2, 4 and 6 mg lovastatin/kg BW, respectively. ^1^ Difference: Difference in the intensity between the comparison treatment groups.

## Supplementary Materials

The following are available online at https://www.mdpi.com/2076-2615/10/1/72/s1, Figure S1: Differentially expressed proteins in the *longissimus thoracis et lumborum* muscle of goats supplemented naturally produced lovastatin/kg body weight.
